# Supplementary material for: The opposing forces of shear flow and sphingosine-1-phosphate control marginal zone B cell shuttling
Source: Nat Commun. 2017 Dec 22;8:2261. doi: 10.1038/s41467-017-02482-4 (PMC5741619; doi:10.1038/s41467-017-02482-4)
Supplement: Supplementary file 1 — Supplementary Information [file 41467_2017_2482_MOESM1_ESM.pdf]

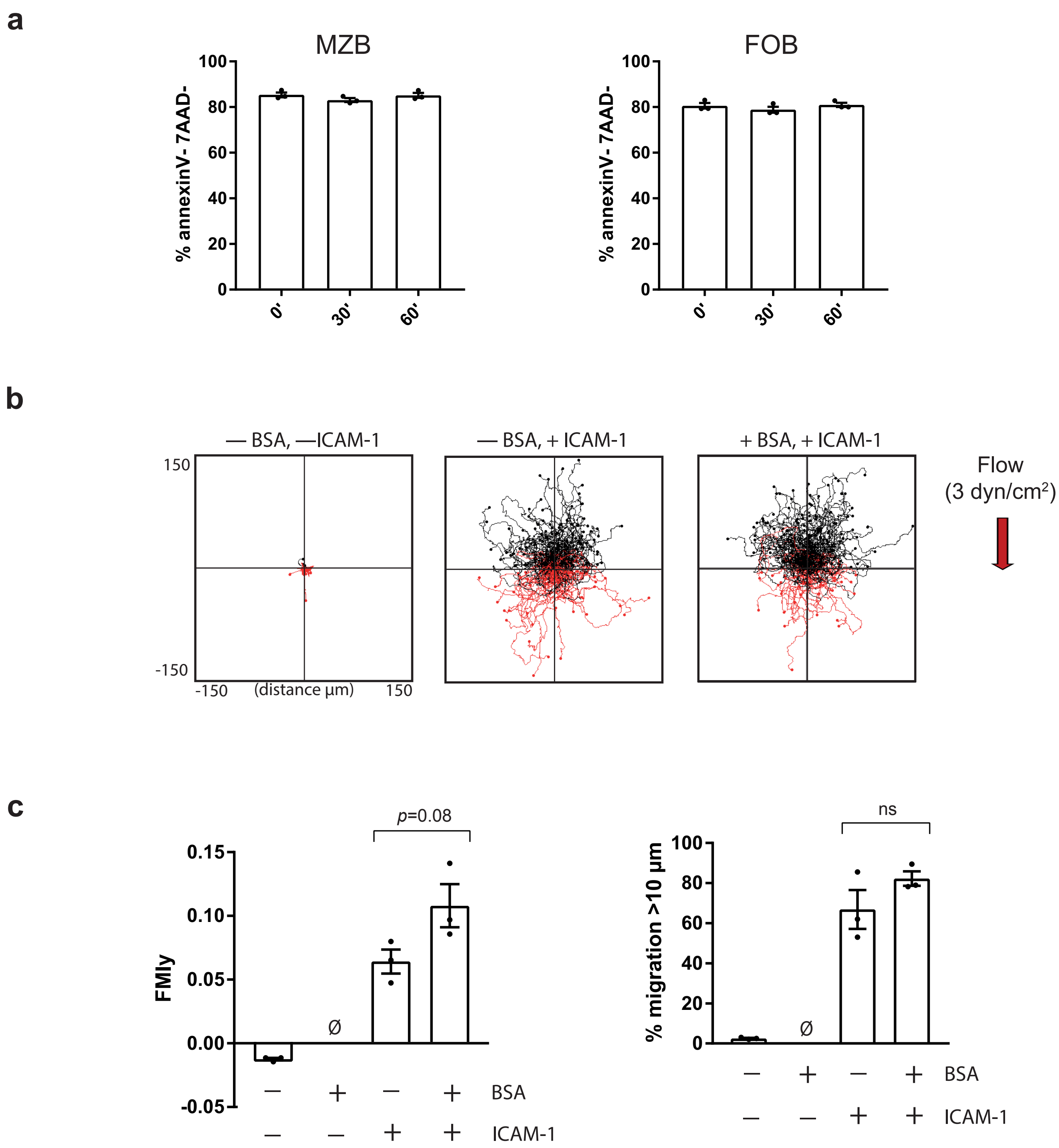

Supplementary Figure 1: Effects of omitting ICAM-1 from slide coatings or of blocking slides with an irrelevant protein BSA. (a) Viability of purified marginal zone B cells (MZB) (left) and follicular B cells (FOB) (right) shown as the percentage of annexinV$^-$ 7AAD$^-$ cells of total cells at the indicated time points (minutes). For flow cytometry gating strategy, see Supplementary Fig. 2a,b. Data are from 1 experiment with 3 mice. Bars show mean ±SEM. **(b)** Representative track plots are shown for MZB migration under flow (3 dyn cm^{-2}) on slides either not coated with ICAM-1 (left), coated with ICAM-1 (5 µg ml^{-1}) but not blocked with BSA (middle), or coated with ICAM-1 and blocked with BSA (2%) (right; standard condition). **(c)** Quantification of migration index (left) and of the % of cells that displace more than 10 µm (right). For the second condition (no ICAM-1 coating but with BSA blocking), almost all cells washed off and were therefore not quantified (shown as Ø). Graphs show different parameters from the same experiment; bars show mean ±SEM; *p* value and ns= not significant by *t*-test. On one graph, per condition, each symbol represents a result from an individual mouse. Data are from 1 experiment with 3 mice.

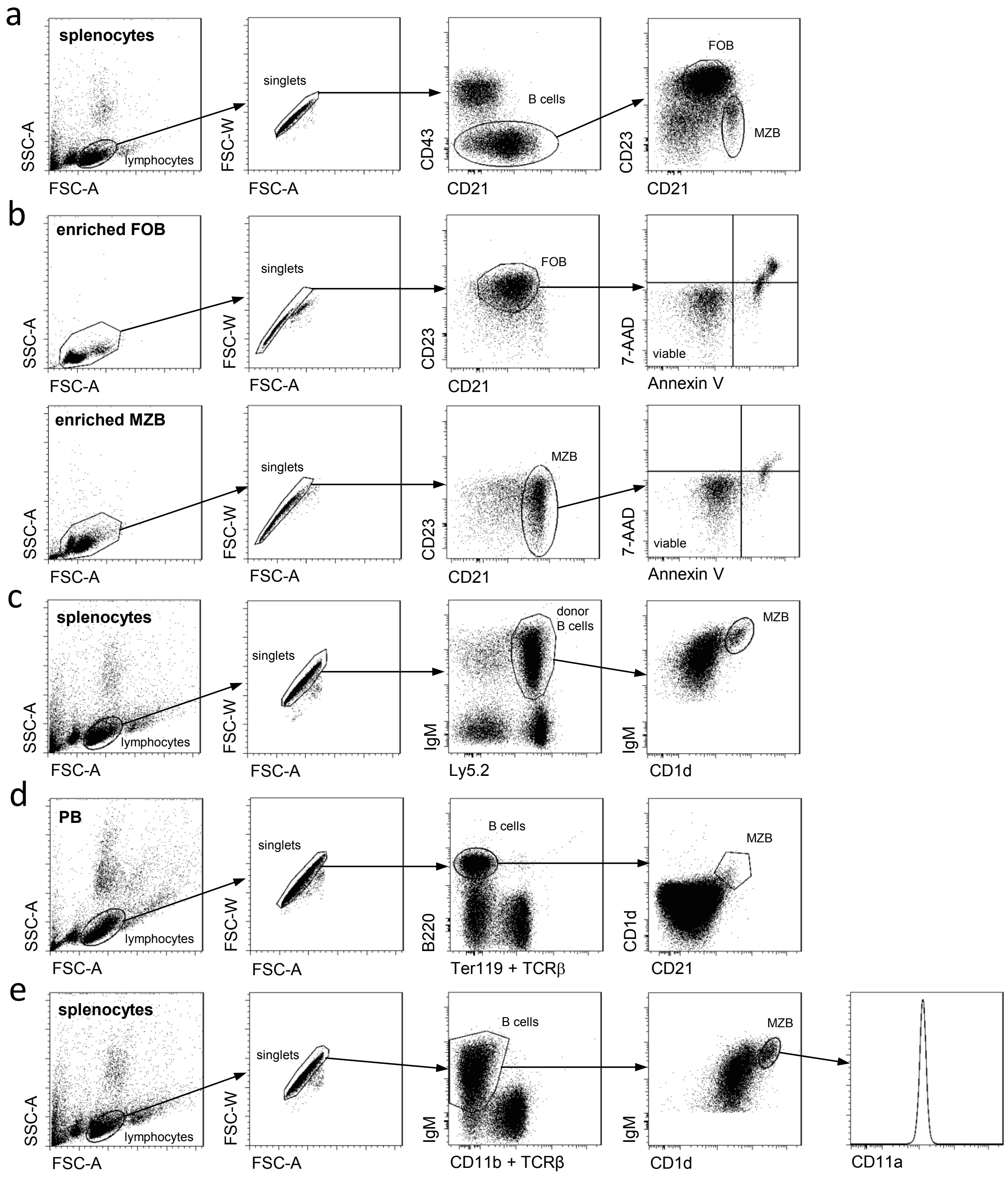

Supplementary Figure 2. Flow cytometry gating strategies. (a) FACS sort of MZB ($CD43^{-}$ $CD23^{lo}$ $CD21^{hi}$) or FOB ($CD43^{-}$ $CD23^{hi}$ $CD21^{lo}$) for in vitro studies in Supplementary Figure 1. **(b)** Viability (% Annexin V^{-}/7-AAD^{-}) of MZB and FOB in Supplementary Figure 1a. **(c)** Analysis of $Ly5.2^{+}$ MZB frequencies in bone marrow chimeras (% IgM^{hi} $CD1d^{hi}$ of $Ly5.2^{+}$ IgM^{+} donor B cells) in Supplementary Figure 6b. **(d)** Detection of MZB ($B220^{+}$ $CD21^{+}$ $CD1d^{+}$) in peripheral blood (PB) of *Arhgef6*$^{-/-}$ mice in Supplementary Figure 6c. **(e)** Integrin expression on MZB (IgM^{hi} $CD1d^{hi}$) shown in Supplementary Figure 7b.

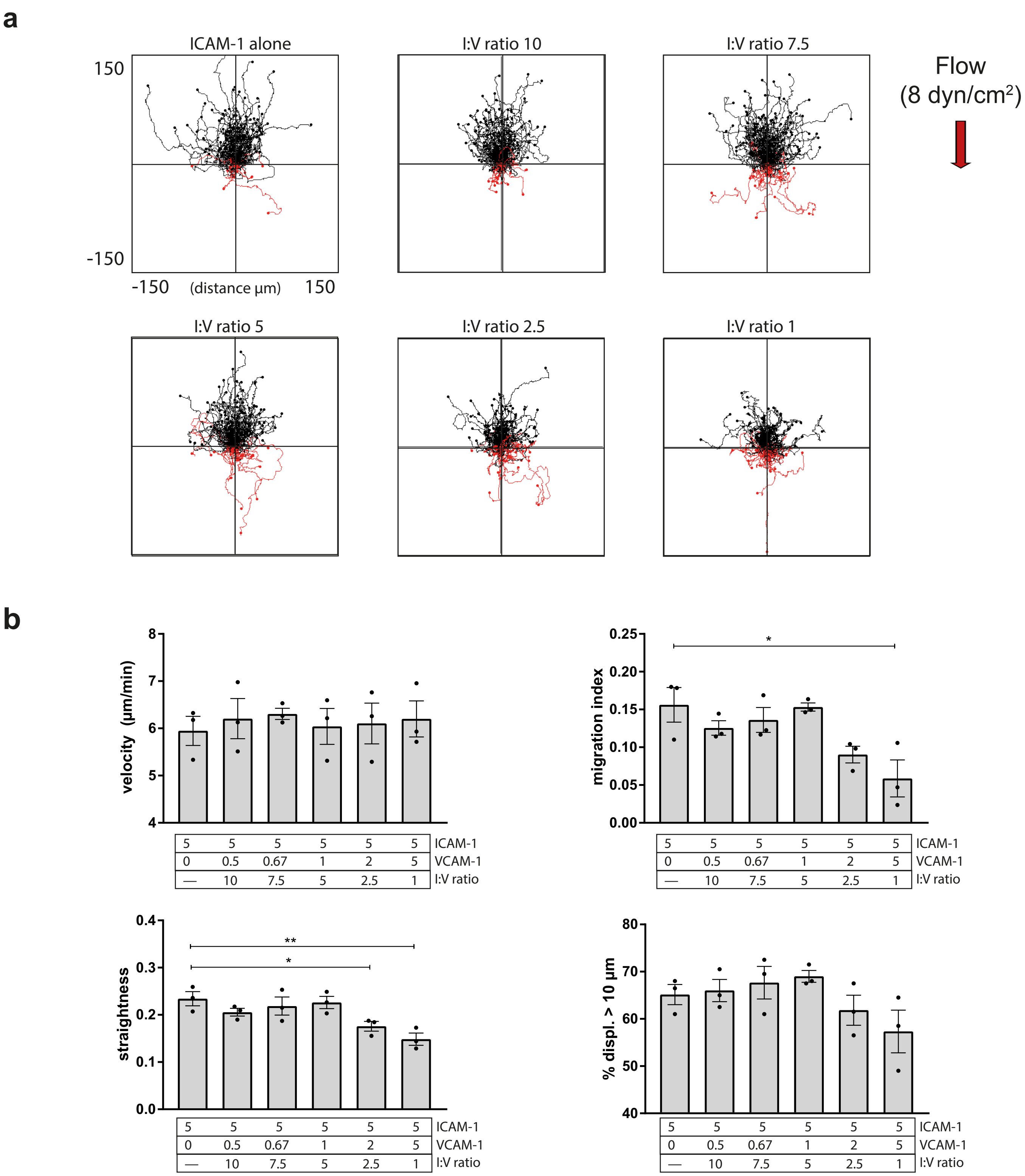

Supplementary Figure 3: Titration of VCAM-1 while holding ICAM-1 concentrations constant. (a) Representative track plots are shown for MZB migration under flow (8 dyn cm^{-2}) on slides coated with ICAM-1 alone (5 µg ml^{-1}) or of ICAM-1 and VCAM-1 at the amounts shown on graphs (in µg ml^{-1}) at the indicated ratios. BSA was added to make up totals of coating protein to 10 µg ml^{-1}. **(b)** Quantification of velocity, migration index, straightness, and % of cells that displace more than 10 µm. All 4 graphs show different parameters from the same set of experiments; bars show mean ±SEM; *p<0.05, ** p <0.01 by one-way ANOVA. Data are from 3 experiments, each with MZB cells from 2 wildtype mice pooled for the 6 conditions. On one graph, per condition, each symbol represents a result (average of 100-200 cell tracks each) from the 2 pooled mice.

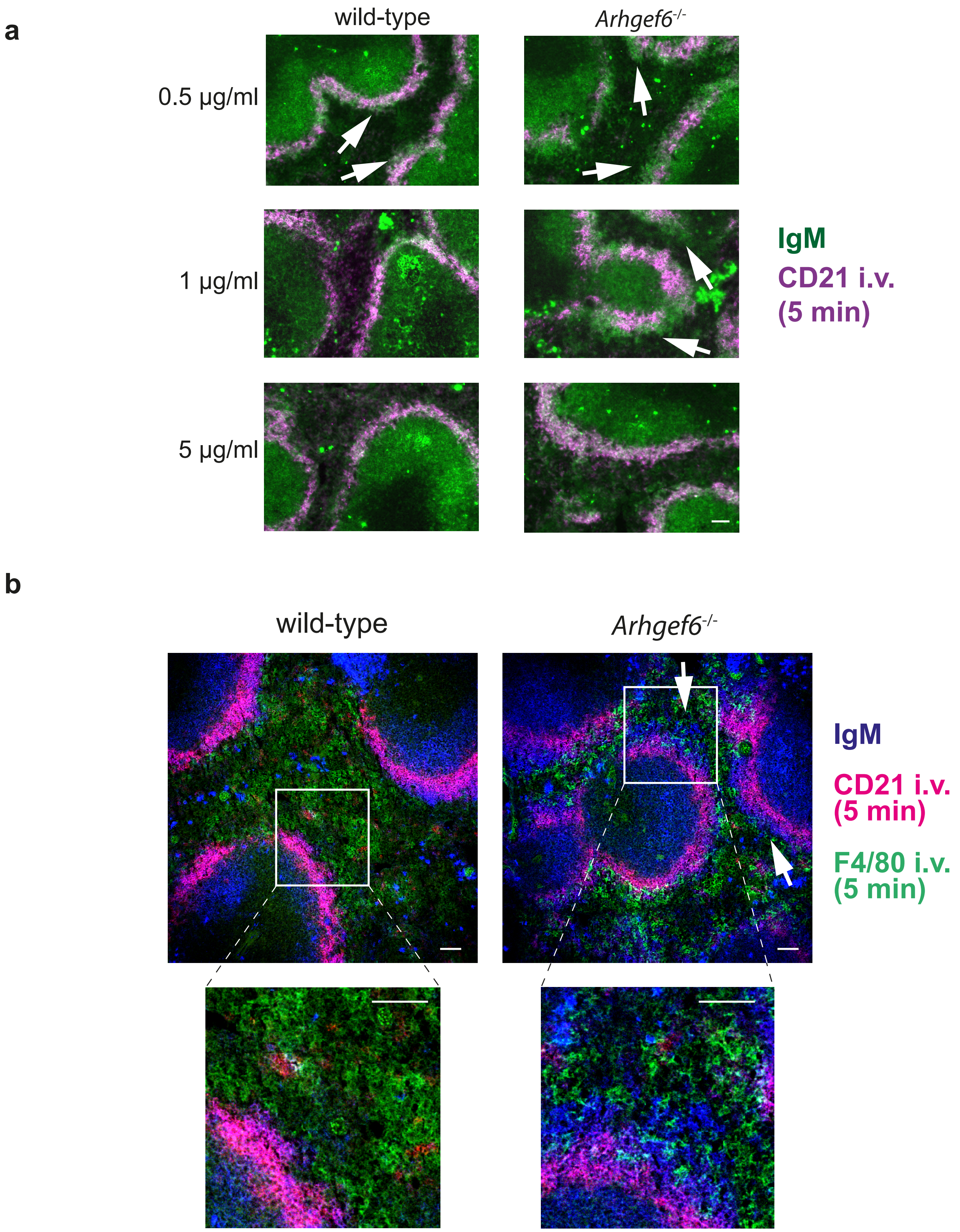

Supplementary 4: Evidence for injected αCD21 as a marker that delimits the marginal zone. (a) Titration of injected αCD21 in wildtype and *Arhgef6*$^{-/-}$ mice. Mice were injected i.v. with αCD21-PE (red) for 5 minutes in the indicated amounts. Spleen sections were also stained for IgM (green). Arrows indicate areas where B cells appear beyond the CD21-labeled marginal zone. WT: n=3, *Arhgef6*$^{-/-}$: n=3, 1 mouse per condition. Scale bar, 50 µm. **(b)** Co-injection of both αCD21 and αF4/80 (1 µg) stains MZB in the marginal zone and red pulp macrophages simultaneously. Co-staining with IgM shows B cells in *Arhgef6*$^{-/-}$ mice beyond the CD21-labeled marginal zone (white arrow). Inset: note the interspersed IgM^{+} and F4/80^{+} cells in *Arhgef6*$^{-/-}$ spleen. Data represent 2 experiments with 1 spleen per genotype. Scale bars, 50 µm.

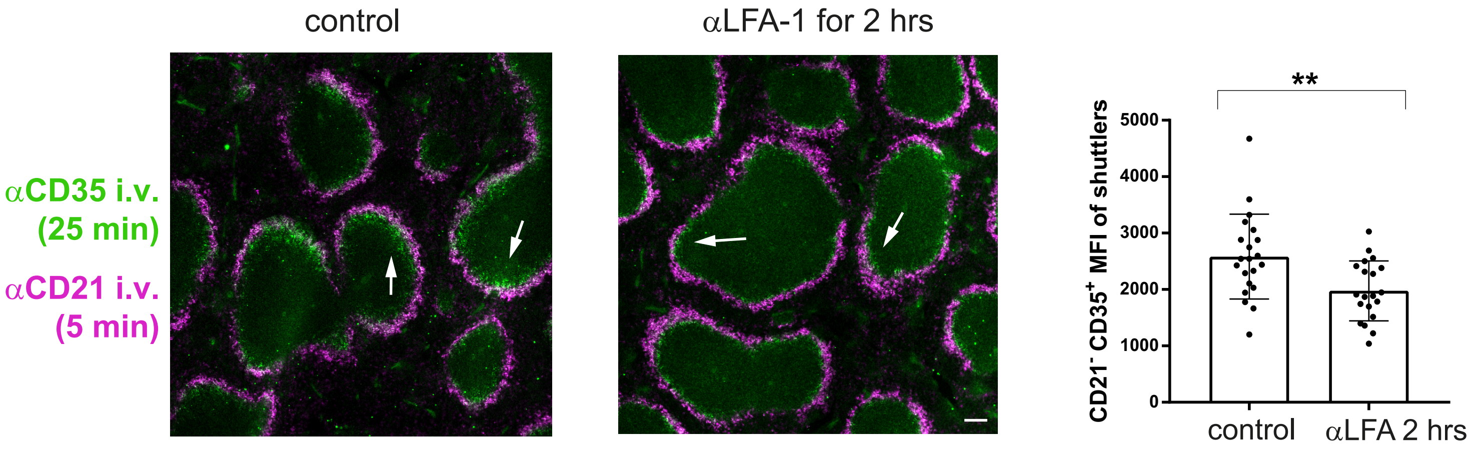

Supplementary Figure 5: LFA-1 blocking injections for 2 hours reduces MZB shuttlers. Immunofluorescence microscopy of $CD35^{+}$ MZB cells shuttling into follicles following 2 hours of i.v. αLFA-1 injection. MZB cells that shuttled into the follicle were identified by double in vivo labeling using i.v. injection of αCD35 for 25 minutes, followed by i.v. injection of αCD21 for 5 minutes. Right panel: quantification of mean gray intensity in a 70 μm band inside of the marginal sinus, determined by co-staining with MAdCAM-1 and CD169 (not shown). Data are representative of 2 additional independent experiments with at least 3 wildtype mice per condition. Symbols in one condition group denote individual follicles, at least 4 per mouse in one experiment. Arrows = $CD35^{+}$ $CD21^{-}$ shuttling MZB inside the follicle. Data are expressed as the mean ±SEM. ** $p < 0.01$ by *t*-test. Scale bar, 100 μm.

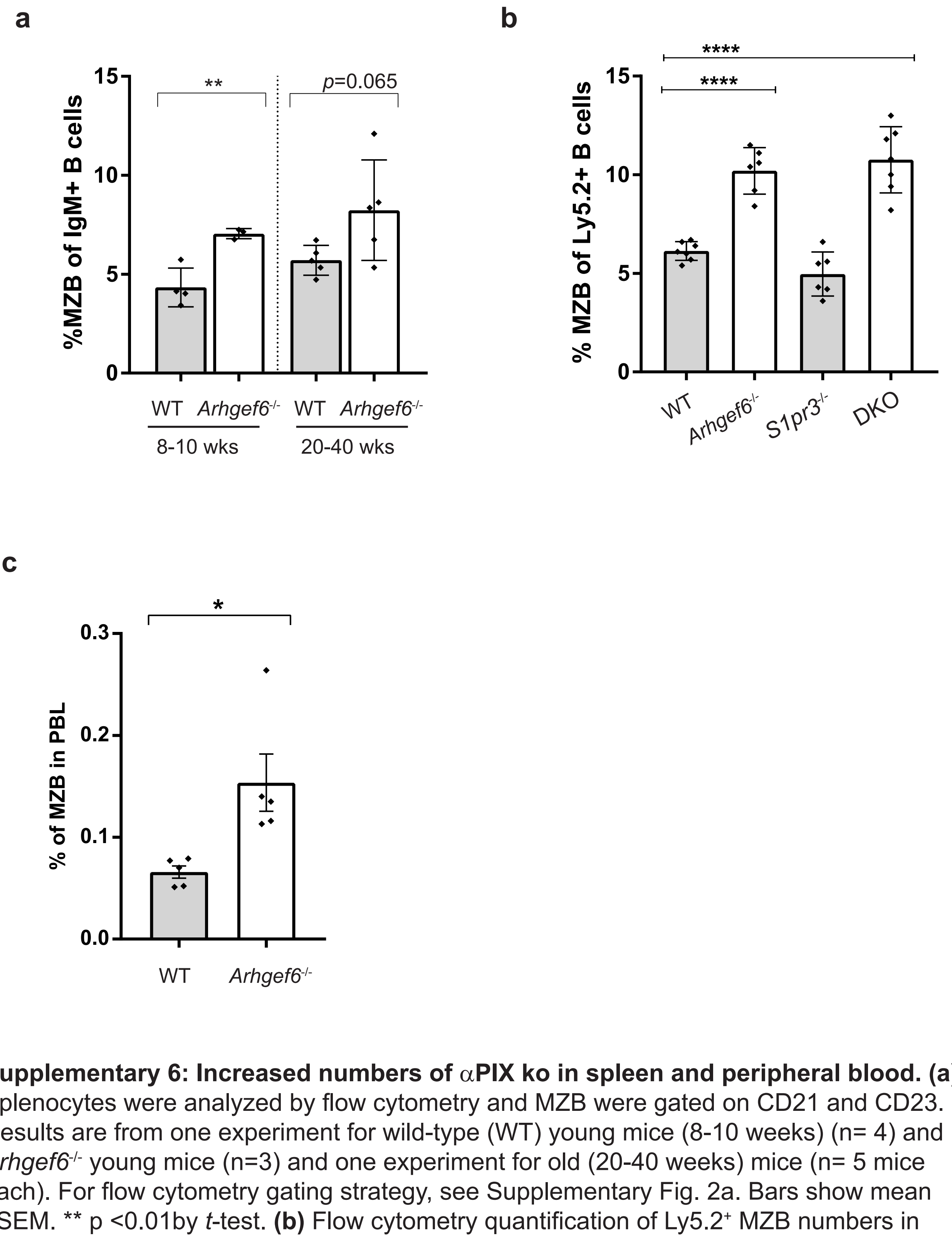

Supplementary 6: Increased numbers of αPIX ko in spleen and peripheral blood. (a) Splenocytes were analyzed by flow cytometry and MZB were gated on CD21 and CD23. Results are from one experiment for wild-type (WT) young mice (8-10 weeks) (n= 4) and *Arhgef6*$^{-/-}$ young mice (n=3) and one experiment for old (20-40 weeks) mice (n= 5 mice each). For flow cytometry gating strategy, see Supplementary Fig. 2a. Bars show mean ±SEM. ** p <0.01by *t*-test. **(b)** Flow cytometry quantification of Ly5.2^{+} MZB numbers in Ly5.1^{+} chimeric mice (WT, *Arhgef6*$^{-/-}$, *S1pr3*$^{-/-}$: n=6; *Arhgef6*$^{-/-,}$ *S1pr3*$^{-/-}$ (DKO): n=7). For flow cytometry gating strategy, see Supplementary Fig. 2c. Bars show mean ±SEM. **** p <0.001 by one-way ANOVA. **(c)** Blood leukocytes from wild-type (WT) and *Arhgef6*$^{-/-}$ mice were analyzed by flow cytometry. For flow cytometry gating strategy, see Supplementary Fig. 2d. Results are from 1 experiment with 4 mice per genotype. Bars show mean ±SEM. * p <0.05 by t-test.

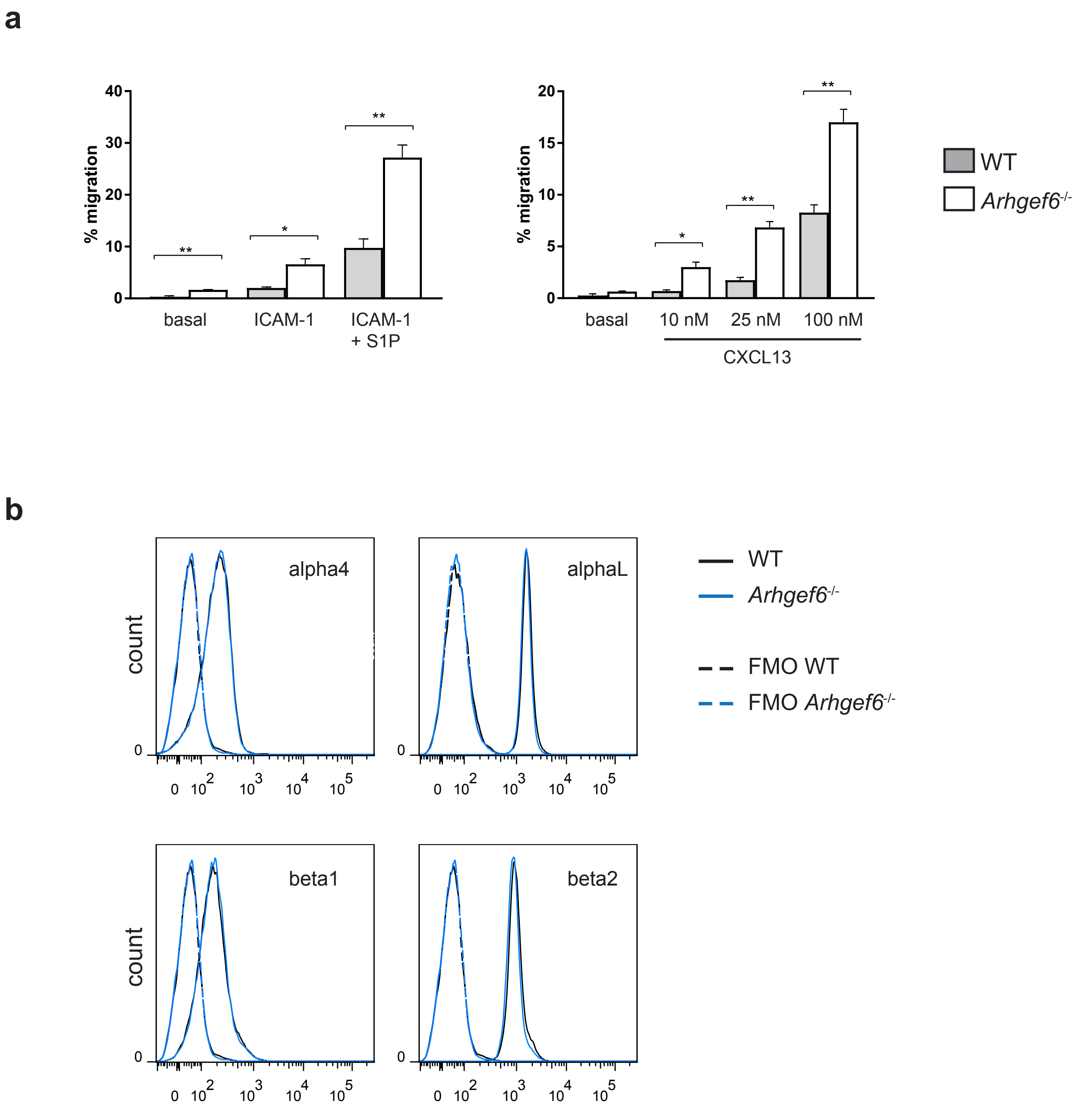

Supplementary 7: Migration of and integrin expression on *Arhgef6*$^{-/-}$ MZB. (a) Increased migration of *Arhgef6*$^{-/-}$ MZB in transwells in all conditions. Left: Transwell migration to S1P through ICAM-1-coated membranes. Right: Transwell migration to CXCL13 in the indicated amounts. Data are representative of 5 experiments with 1 mouse per genotype each. Bars show mean +SEM; **p*<0.05, ** *p* <0.01 by t-test. **(b)** Normal expression of LFA-1 (αLβ2) and VLA-4 (α4β1) integrins on *Arhgef6*$^{-/-}$ MZB analyzed by flow cytometry. FMO = fluorescent minus one controls. For flow cytometry gating strategy, see Supplementary Fig. 2e. Data represent 1 experiment with 3 mice.

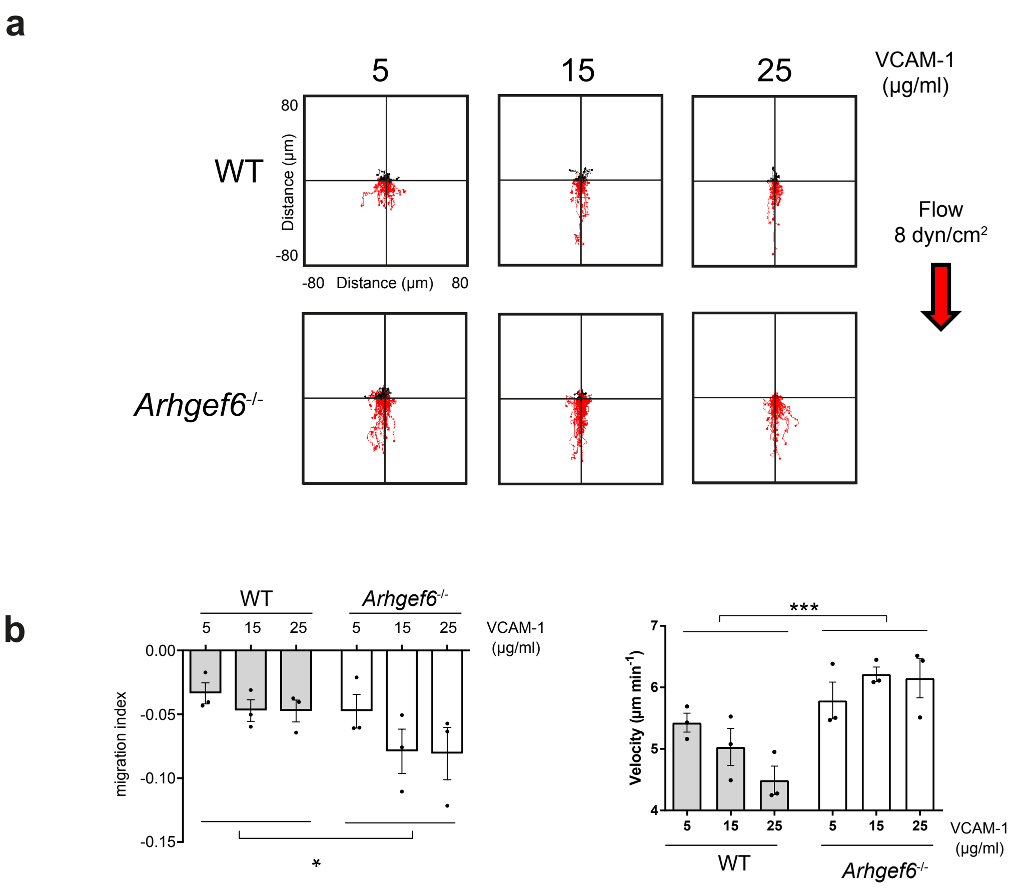

Supplementary 8: Migration of *Arhgef6*$^{-/-}$ MZB under flow on high VCAM-1 concentrations. (a) Representative track plots of wild-type (WT) and *Arhgef6*$^{-/-}$ MZB cells migrating on increasing VCAM-1 concentrations (5, 15, or 25 µg ml^{-1} VCAM-1 as indicated) under flow (8 dyn cm^{-2}). **(b)** Quantification of velocity and migration index for track plots. Bars show mean ±SEM. *p<0.05, *** p <0.001, by two-way ANOVA. 3 mice per genotype in 3 experiments with 1 genotype each.

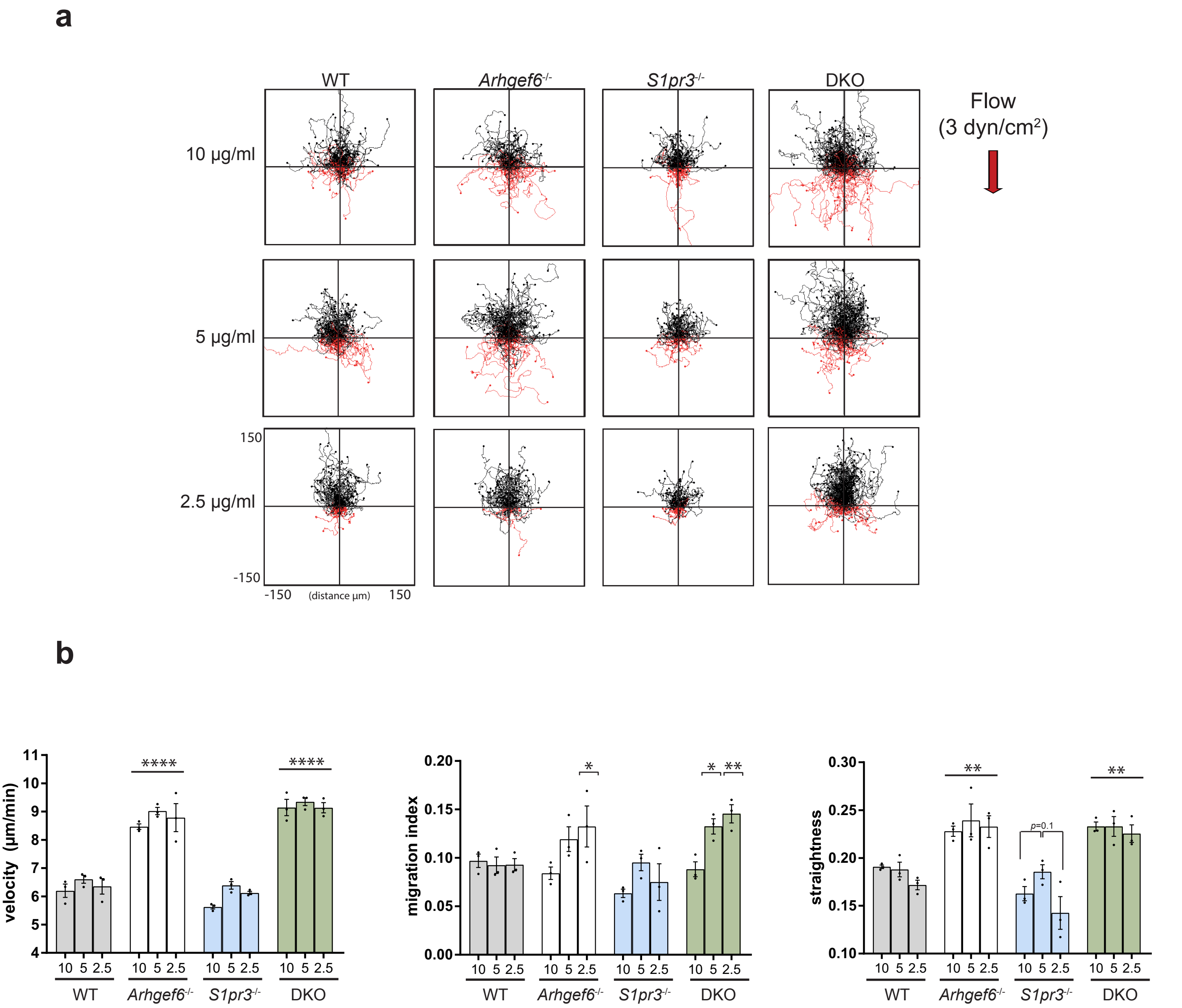

Supplementary 9: Titration of ICAM-1 in MZB flow migration. (a) Representative track plots are shown for migration under flow (3 dyn cm^{-2}) of wild-type (WT), *Arhgef6*$^{-/-}$, *S1pr3*$^{-/-}$, and *Arhgef6*$^{-/-}$ *S1pr3*$^{-/-}$ (DKO) MZB on 10, 5, and 2.5 µg ml^{-1} ICAM-1. **(b)** Quantification of velocity, migration index, and straightness. All 3 graphs show different parameters from the same set of experiments; bars show mean ±SEM. *p<0.05, ** p <0.01, **** p <0.0001, by two-way ANOVA for velocity and straightness. For migration index, Dunnett's post-hoc tests relative to 10 µg ml^{-1} are shown; for straightness of *S1pr3*$^{-/-}$, t-tests relative to 5 µg ml^{-1} are shown. Data are from 6 experiments with 2 mice each, 3 mice total per genotype. Symbols in one genotype group denote the 3 mouse replicates (each symbol is the average of 100-200 cells).

Supplementary Note 1:

It was unexpected that the five minute injection of αCD21 would mark MZB cells inside the marginal zone but not directly outside it, as there is no tight endothelial barrier to the red pulp as there is to the follicle. To validate the approach, we titrated the amounts of injected αCD21 to determine the extent of cells stained in five minutes. Using a lesser amount of αCD21 (0.5 µg), we detected areas of IgM-positive cells outside the CD21-marked zone. With more antibody (1 µg or 5 µg), these areas were no longer visible as the cells were fully stained on CD21 (Supplementary Fig. 4a). We also tested these amounts of αCD21 in *αPIX* knockout (ko) mouse, which show strongly increased numbers of MZB outside the marginal zone. The same effect was evident, albeit with higher amounts of αCD21: the *αPIX* ko MZB cells at the outside of the CD21 border were only stained when 5 µg of αCD21 was used (Supplementary Fig. 4a). These results show that suboptimal amounts of αCD21 are preferentially taken up by staining of MZB cells in the marginal zone, and that MZB cells located at the border of the marginal zone and the red pulp and beyond are not stained unless the amount of αCD21 is increased.

We then co-injected mice with αCD21 (1 µg) and αF4/80, which stains red pulp macrophages, and observed that the αF4/80 antibody could flow through the marginal zone to the red pulp and stain macrophages there, while the αCD21 antibody was taken up by MZB in the marginal zone (Supplementary Fig. 4b). However, in *αPIX* ko spleens, the MZB outside the marginal zone remained unstained by αCD21, showing that the αCD21 at this amount does not stain all MZB located close to but outside the marginal zone. In the *αPIX* ko spleen, the intermingling of the IgM cells with F4/80+ red pulp macrophages revealed that the *αPIX* ko MZB are localized in the red pulp (Supplementary Fig. 4b, insets). It is unclear why the antibody would not diffuse fully around the cells just outside the marginal zone if there is no physical barrier. One possible explanation is that VCAM-1 levels are high enough in this area to cause MZB cells to adhere strongly. This could inhibit coating of the cells with the CD21 antibody, as they would be constrained from rotating or moving around. With suboptimal amounts, the MZB cells that can move freely in the antibody suspension would have an advantage in becoming stained. Nevertheless, the method was effective for delineating the marginal zone and enabling the quantification of MZB cells outside it.
