# Supplementary material for: The opposing forces of shear flow and sphingosine-1-phosphate control marginal zone B cell shuttling
Source: Nat Commun. 2017 Dec 22;8:2261. doi: 10.1038/s41467-017-02482-4 (PMC5741619; doi:10.1038/s41467-017-02482-4)
Supplement: Supplementary file 3 — Description of Additional Supplementary Files [file 41467_2017_2482_MOESM3_ESM.pdf]

Description of Additional Supplementary Files

File Name: Supplementary Movie 1
Description: Wild-type MZBs migrate up the flow.

File Name: Supplementary Movie 2
Description: *Arhgef6*$^{-/-}$ MZBs migrate faster than wild-type up the flow, thus producing longer tracks.

File Name: Supplementary Movie 3
Description: *S1pr3*$^{-/-}$ MZB migration up the flow shows moderate decrease in straightness.

File Name: Supplementary Movie 4
Description: *Arhgef6*$^{-/-}$ *S1pr3*$^{-/-}$ MZBs migrate faster than wild-type up the flow, thus producing longer tracks.

File Name: Supplementary Movie 5
Description: Wild-type MZBs treated with S1P migrate less effectively up the flow.

File Name: Supplementary Movie 6
Description: *Arhgef6*$^{-/-}$ MZBs treated with S1P migrate less effectively up the flow.

File Name: Supplementary Movie 7
Description: *S1pr3*$^{-/-}$ MZB migration up the flow unaffected by S1P.

File Name: Supplementary Movie 8
Description: *Arhgef6*$^{-/-}$ *S1pr3*$^{-/-}$ MZB migration up the flow unaffected by S1P.
